# Reduced graphene oxide membrane as supporting film for high-resolution cryo-EM

**DOI:** 10.52601/bpr.2021.210007

**Published:** 2021-06-30

**Authors:** Nan Liu, Liming Zheng, Jie Xu, Jia Wang, Cuixia Hu, Jun Lan, Xing Zhang, Jincan Zhang, Kui Xu, Hang Cheng, Zi Yang, Xin Gao, Xinquan Wang, Hailin Peng, Yanan Chen, Hong-Wei Wang

**Affiliations:** 1 Ministry of Education Key Laboratory of Protein Sciences, Beijing Advanced Innovation Center for Structural Biology, School of Life Sciences, Tsinghua University, Beijing 100084, China; 2 Tsinghua-Peking Joint Center for Life Sciences, Tsinghua University, Beijing 100084, China; 3 Center for Nanochemistry, Beijing Science and Engineering Center for Nanocarbons, Beijing National Laboratory for Molecular Sciences, College of Chemistry and Molecular Engineering, Peking University, Beijing 100871, China; 4 Joint Graduate Program of Peking-Tsinghua-NIBS, School of Life Sciences, Tsinghua University, Beijing 100084, China; 5 Academy for Advanced Interdisciplinary Studies, Peking University, Beijing 100871, China; 6 School of Materials Science and Engineering, Tianjin University, Tianjin 300072, China; 7 Beijing Frontier Research Center for Biological Structures, Tsinghua University, Beijing 100084, China

**Keywords:** Reduced graphene oxide, Cryo-EM, 3D reconstruction, Air–water interface

## Abstract

Although single-particle cryogenic electron microscopy (cryo-EM) has been applied extensively for elucidating many crucial biological mechanisms at the molecular level, this technique still faces critical challenges, the major one of which is to prepare the high-quality cryo-EM specimen. Aiming to achieve a more reproducible and efficient cryo-EM specimen preparation, novel supporting films including graphene-based two-dimensional materials have been explored in recent years. Here we report a robust and simple method to fabricate EM grids coated with single- or few-layer reduced graphene oxide (RGO) membrane in large batch for high-resolution cryo-EM structural determination. The RGO membrane has decreased interlayer space and enhanced electrical conductivity in comparison to regular graphene oxide (GO) membrane. Moreover, we found that the RGO supporting film exhibited nice particle-absorption ability, thus avoiding the air–water interface problem. More importantly, we found that the RGO supporting film is particularly useful in cryo-EM reconstruction of sub-100-kDa biomolecules at near-atomic resolution, as exemplified by the study of RBD-ACE2 complex and other small protein molecules. We envision that the RGO membranes can be used as a robust graphene-based supporting film in cryo-EM specimen preparation.

## INTRODUCTION

Single-particle cryo-EM, without the requirement of crystallization, has become a major method to solve structures of biological macromolecules at near-atomic resolution benefiting from its recent advances in both software and hardware (Cheng 2015, 2018; Li *et al*. 2013; Scheres 2012; Wu *et al*. 2016). The more recent breakthroughs even pushed the resolution to genuine atomic level (Nakane *et al*. 2020; Yip *et al*. 2020). Furthermore, the method enables the analysis of heterogeneity to solve multiple conformers from one single cryo-EM dataset (Scheres 2012). While cryo-EM has become one of the most powerful methods in structural biology, a major bottleneck limiting the method’s general application is the poor reproducibility and controllability to prepare cryo-EM specimen (Armstrong *et al*. 2020; Glaeser 2016, 2018). The conventional and most popularly used cryo-EM specimen preparation procedure is to apply a droplet of solution containing target macromolecules onto an EM grid coated with holey carbon film, to blot the grid with filter paper and then to plunge-freeze the grid into liquid nitrogen temperature to form a very thin vitreous ice embedding the macromolecules (Dubochet *et al*. 1982; Grassucci *et al*. 2007). During the blotting process to form thin liquid film, biomolecular particles with protein components tend to be absorbed onto the air–water interface, causing denaturation and preferred orientation (Glaeser 2018; Glaeser and Han 2017; Noble *et al*. 2018). The adsorption of molecules to the air–water interface was hypothesized to be the main reason of low yield in cryo-EM specimen preparation (D'Imprima *et al*. 2019; Noble *et al*. 2018). To overcome this problem, thin films of various materials have been developed to support the biological macromolecules over holey grids. The most frequently used thin film is continuous amorphous carbon, which contributes to strong background noise, aggravate charging effect and beam-induced motion due to its poor electrical conductivity when imaged under electron microscopy (Russo and Passmore 2014a, b; Zheng *et al*. 2020).

Graphene (Geim and Novoselov 2007), a two-dimensional nano-material with the superior properties of ultrahigh electrical/thermal conductivity (Balandin *et al*. 2008; Chen *et al*. 2008), mechanical strength (Lee *et al*. 2008) and low background noise (Russo and Passmore 2014a), is considered as an ideal supporting film for cryo-EM specimen preparation. Graphene membrane as well as its derivatives, such as functionalized graphene (D'Imprima *et al*. 2019; Han *et al*. 2020; Liu *et al*. 2019; Naydenova *et al*. 2019; Russo and Passmore 2014a), have been reported to help successfully determining high-resolution reconstruction of multiple macromolecular structures by cryo-EM. The synthesis of single-crystalline graphene with large area, however, due to its high technical and resource demand (Lin *et al*. 2016), is difficult to be established in regular laboratories focusing on biology. Moreover, the preparation of CVD-prepared or commercially available graphene onto EM grids normally involves many chemical reagents and is hard to avoid contaminations (Han *et al*. 2020; Regan *et al*. 2010; Zhang *et al*. 2017). On the other hand, graphene oxide (GO) containing plenty of functional groups like carboxyl or epoxy (Pantelic *et al*. 2010) can be easily generated by oxidizing graphite, therefore has been explored as supporting film for cryo-EM specimen preparation (Benjamin *et al*. 2016; Palovcak *et al*. 2018; Pantelic *et al*. 2010; Wang *et al*. 2020; Wilson *et al*. 2009). However, GO membrane is electrical insulative (Jung *et al*. 2008; Wang *et al*. 2018), which may cause charging accumulation in the irradiated region (Egerton *et al*. 2004). In addition, the space of interlayer in multiple-layer GO films is larger than that of multi-layer graphene because of the abundant functional groups and encapsulated solvent molecules in the GO films, introducing extra background noise (Moon *et al*. 2010; Qiu *et al*. 2015).

Herein, we develop a facile and robust strategy to use reduced graphene oxide (RGO) membrane as supporting substrate for cryo-EM specimen preparation. Compared with GO, RGO contained fewer functional groups with decreased interlayer space and better electric conductivity. Notably, the RGO membrane enabled nice absorption of target biomolecules and high-resolution cryo-EM reconstruction. Several sub-100 kDa biomolecules exhibited nice contrast on RGO membrane, and we successfully solved the structure of SARS-CoV2 RBD-ACE2 complex at 2.8 Å resolution. In our practice, the RGO membrane seemed particularly useful for cryo-EM analysis of relatively small protein molecules in low concentration.

## RESULTS

### Fabrication and characterization of RGO grids

**Figure 1:**
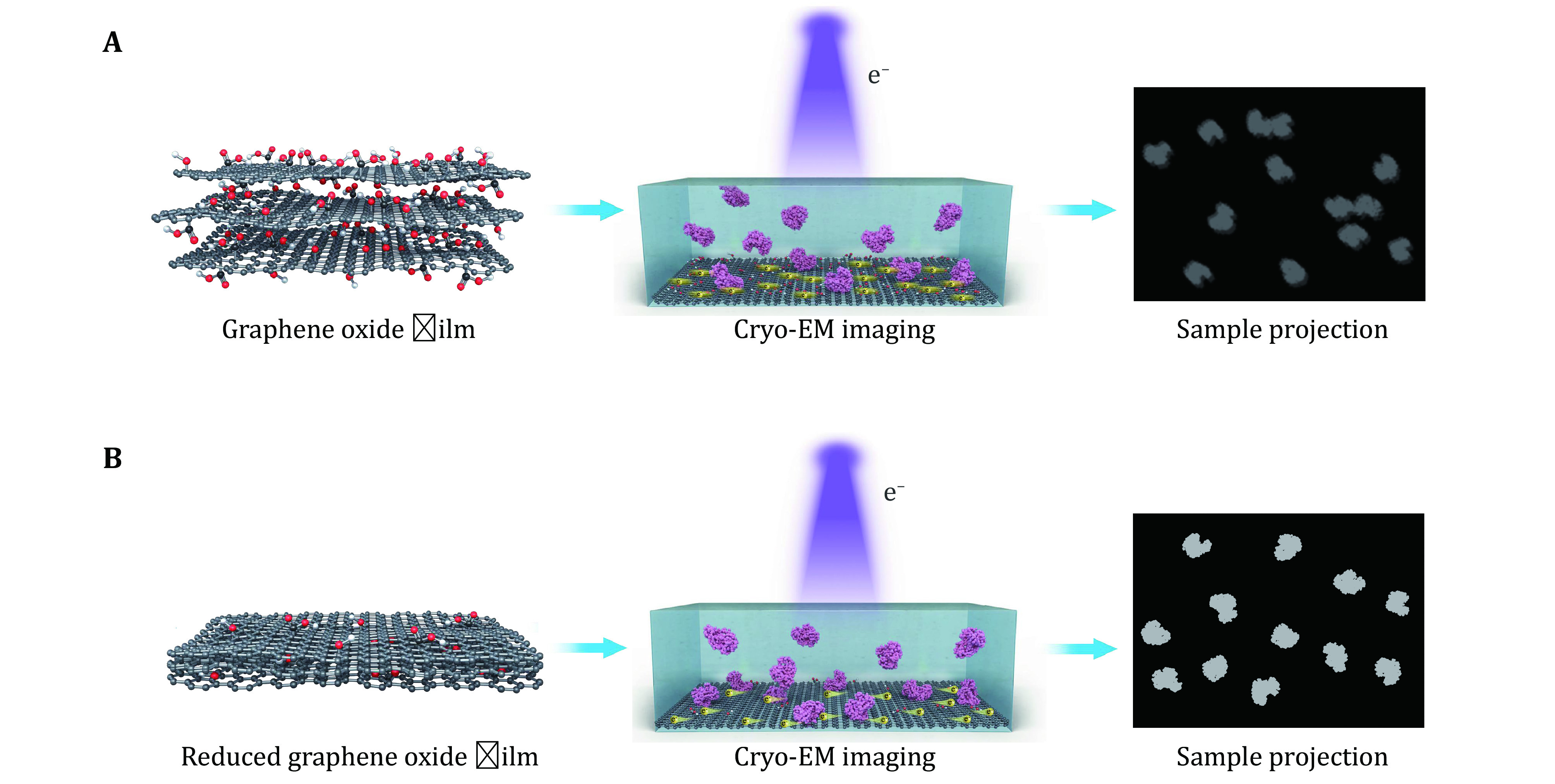
Schematic diagram of graphene oxide (**A**) and reduced graphene oxide (**B**) for single-particle cryo-EM analysis

Graphene oxide (GO) could be produced by improved Hummers’ method (Marcano *et al*. 2010) or commercially purchased, and dispersed in methanol/water solution. The GO layers were firstly coated onto holey-carbon EM grids following the previously reported method (Wang *et al*. 2020). The grids were then baked in an atmosphere of hydrogen/argon to reduce the GO membranes into RGO (Fig. 1 and supplementary Fig. S1). RGO grids can be simply fabricated in large batch and we can normally prepare several hundred RGO grids at a time.

**Figure 2:**
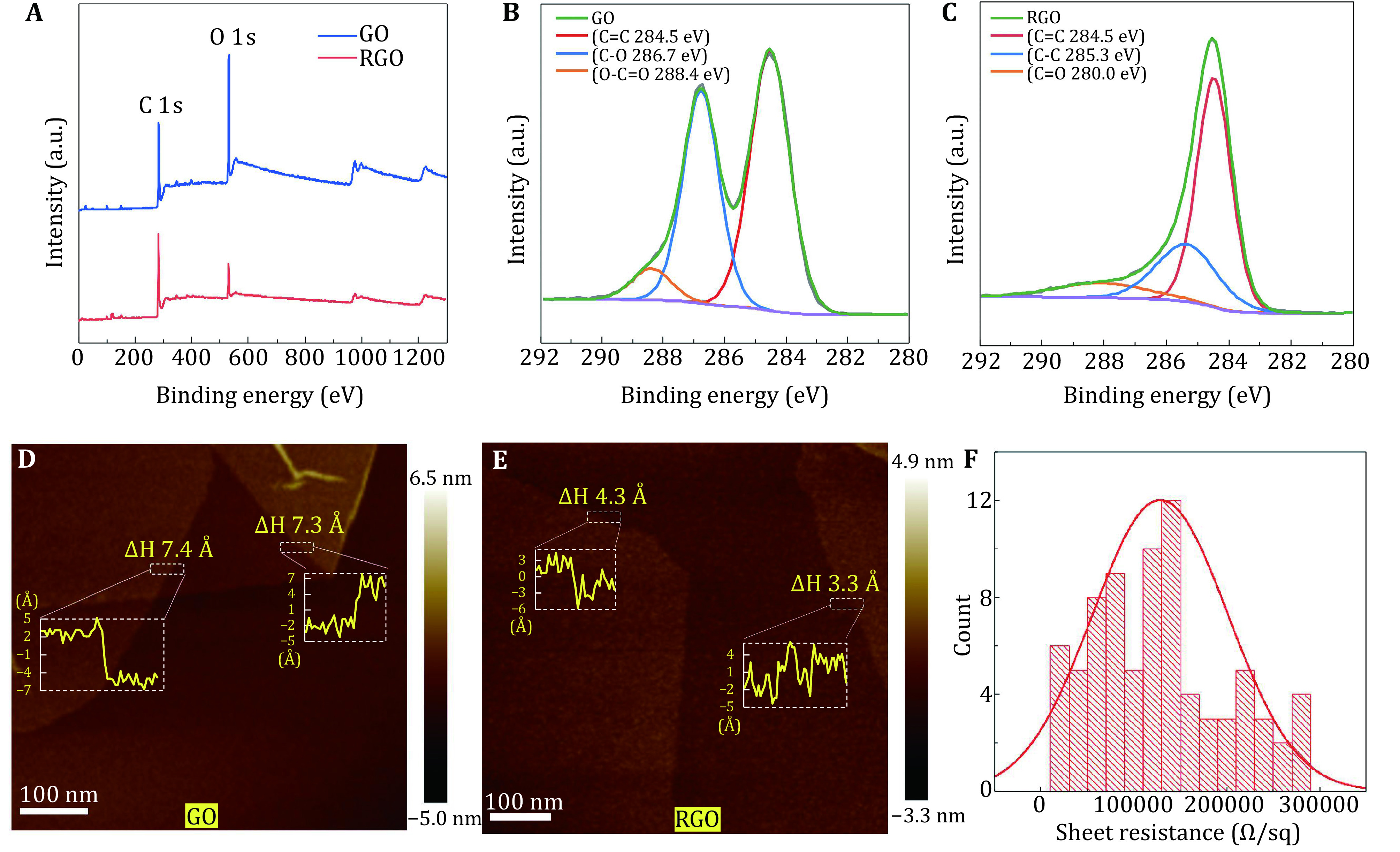
Characterization of reduced graphene oxide membrane. **A** XPS spectra of graphene oxide (GO) and reduced graphene oxide (RGO). Energy binding peaks assigned to C1s and O1s of 284.4 eV and 532.8 eV were labeled. **B** High-resolution XPS C1s spectra of graphene oxide. **C** High-resolution XPS C1s spectra of reduced graphene oxide. **D** Interlayer space of graphene oxide membrane measured by AFM. The measured regions and corresponding height plots were windowed by rectangles. **E** Interlayer space of reduced graphene oxide membrane measured by AFM. **F** Sheet-resistance histogram of reduced graphene oxide membrane

We characterized the reduction efficiency of functional groups on GO by X-ray photoelectron spectroscopy (XPS) and found that the O1s peak intensity decreased after reduction treatment (Fig. 2A and supplementary Fig. S2). The atomic ratio (O1s/C1s) of RGO was reduced to 0.16 from 0.38 of GO, consistent with the reported results by chemical graphitization (Moon *et al*. 2010). We further analyzed the high-resolution spectra of C1s. The signals of C–O (binding energy 286.7 eV) and O–C=O (288.4 eV) bonds which were evident in GO entirely disappeared in RGO, where the sp^2^ carbon of C=C (284.5 eV) and C–C (285.3 eV) dominated the bonding species (Fig. 2B and 2C). Accordingly, the peak area ratio of oxygen functional groups in RGO was down to 0.06, which was significantly decreased in comparison with that (~0.47) of GO. The spectrum analysis demonstrated that a large number of oxygen-containing functional groups on graphene oxide layers had been successfully eliminated by the reduction treatment.

In order to measure the interlayer distance of GO and RGO membranes, we coated both samples on fresh mica plate and examined them by atomic force microscopy (AFM). The interlayer space can be measured by the height profile of two neighboring layers. The space of RGO was 0.3–0.4 nm (Fig. 2E), consistent with previous reports (Lian *et al*. 2018; Moon *et al*. 2010), about half of that of GO with over 0.7 nm (Fig. 2D) (Buchsteiner *et al*. 2006; Lian *et al*. 2018; You *et al*. 2013). The interlayer space reduction can be explained by two reasons. First, the branching functional groups covalently bound onto GO layers stretch the interlayer space, while the layers of RGO stack more tightly due to the lack of branching functional groups. Second, the GO membranes with active functional groups may attract small molecules like water, further expanding the interlayer space (Lian *et al*. 2018). The interlayer space of RGO is similar to that of the CVD-prepared multilayer graphene membranes of ~0.35 nm, further indicating a similar structure of RGO to graphene. We further tested the electrical conductivity of GO and RGO to see which one is more conductive as the supporting material in EM. We verified that the GO sheet was electrically insulative (Qiu *et al*. 2015; Wang *et al*. 2018) and its sheet-resistance exceeded the measuring range in our characterization. In contrast, the sheet-resistance of RGO was dramatically reduced (~10^5^ Ω per square), and the lowest resistance can be 20,000 Ω per square (Fig. 2F).

### Characterization of the hydrophilicity and charging effect of RGO membrane

**Figure 3:**
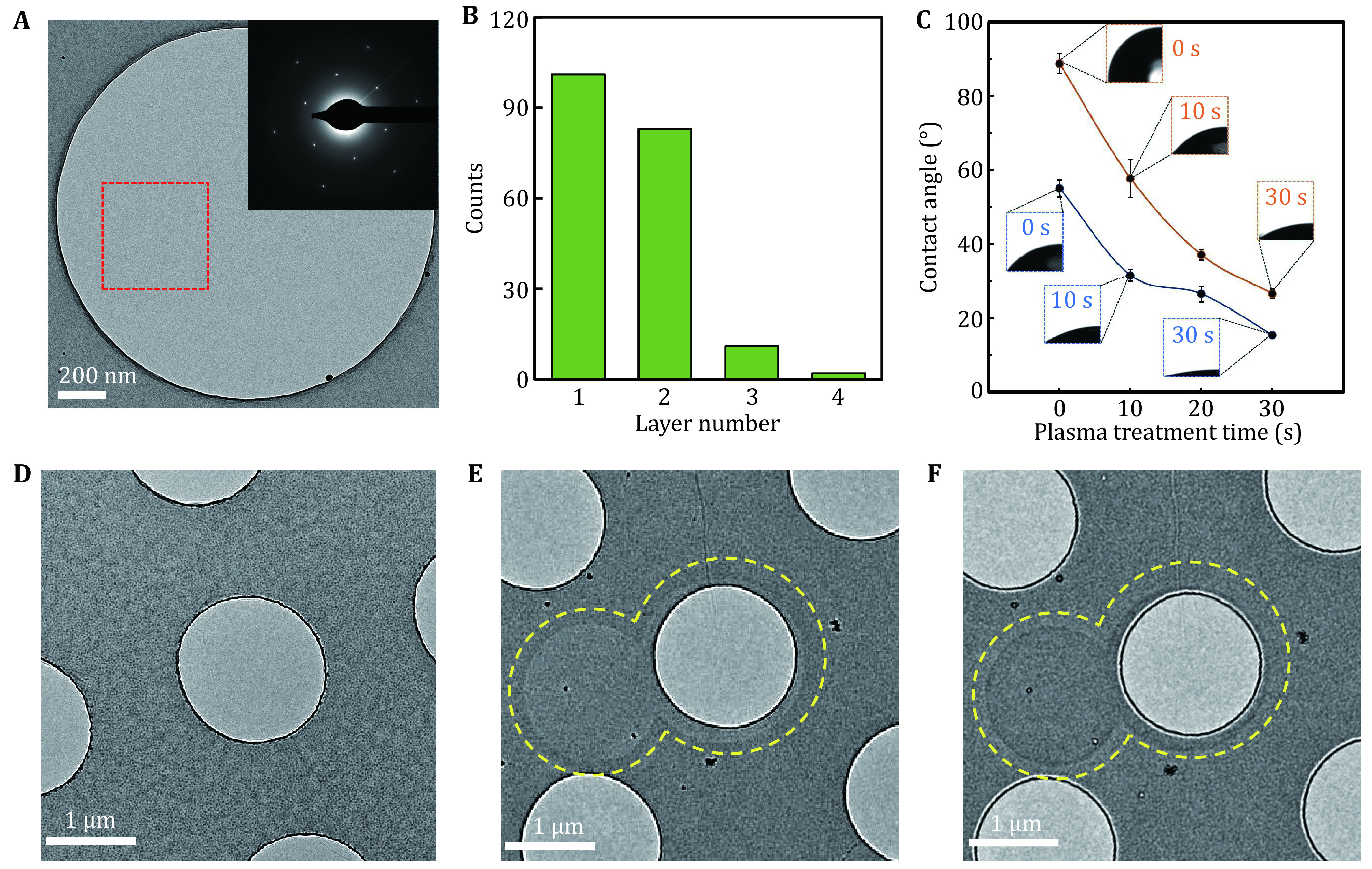
TEM characterization and hydrophilicity of RGO and GO membranes. **A** A representative TEM image of RGO monolayer covering a hole. The inset was the electron diffraction pattern of the selected region labeled by red square. **B** Statistics of graphene layer number across holes on the EM grid. **C** Water contact angles of RGO (orange curve) and GO (dark blue) coated grids after 0, 10, 20 and 30 s low-energy plasma treatment. **D** A low-magnification micrograph of RGO membrane imaged under defocus condition after irradiation of smaller regions at a total dose of ~200 e^−^/Å^2^. **E** A low-magnification micrograph of GO membrane imaged under defocus condition after irradiation of smaller regions (circled by dotted yellow lines) at a total dose of ~200 e^−^/Å^2^. **F** A micrograph of the same area in **E** taken at overfocus condition. Electron beam-induced footprints are visible in **F** and **E**

The coverage of RGO membrane on EM grids prepared as described above was more than 90% and free of contamination (Fig. 3A and supplementary Fig. S3, S4). The reduction procedures barely broke the graphene membrane and kept the layer number of GO as in the initial coating (supplementary Fig. S3). It was ordinary to find single-layer graphene with good crystallinity covering an entire hole, as indicated by the selected area electron-diffraction pattern (Fig. 3A and supplementary Fig. S4). We further evaluated the layer number of graphene across holes and found that more than half of the counted holes were covered by single-layer graphene and ~40% were covered by two-layer graphene (Fig. 3B), which verified that the majority of the holes were covered by single- or few-layer graphene film, thus generating much less background noise than conventional amorphous carbon supporting film. We tested the hydrophilicity of GO and RGO coated grids, by measuring the water contact angle (WCA) after low-energy plasma treatments. As expected, RGO grids with fewer functional groups exhibited larger WCA compared to GO grids, indicating stronger hydrophobicity. However, their WCAs were rapidly decreased when treated with plasma cleaning (Fig. 3C), suitable to be used for cryo-EM specimen preparation.

We analyzed the beam-induced effect of RGO and GO. After electron irradiation with an accumulated dose of ~200 e^−^/Å^2^ at room temperature, the RGO membrane had no visible beam-induced footprints (Fig. 3D), probably due to its improved electrical conductivity. In contrast, there were obvious beam-induced image marks left on the GO membrane after being irradiated with the same dose, exhibiting as “white disks” in de-focused (Fig. 3E) and “black disks” in over-focused micrographs (Fig. 3F). These radiation-induced disks may be caused by charging (Brink *et al*. 1998; Danev *et al*. 2014; Hettler *et al*. 2018) or mass loss effects (Choppin *et al*. 2013; Jiang and Spence 2009). On the electric-insulating GO membranes, charge could be built up by the accumulated electrons, generating a phase contrast that can be observed in out-of-focus micrographs (Fig. 3E and 3F). On the other hand, small molecules and functional groups sandwiched among GO layers are dose-sensitive and susceptible to electron-beam bombardment (Moon *et al*. 2010; Zhang *et al*. 2009), thereby causing decomposition at the exposed regions.

### RGO for Cryo-EM reconstructions

**Figure 4:**
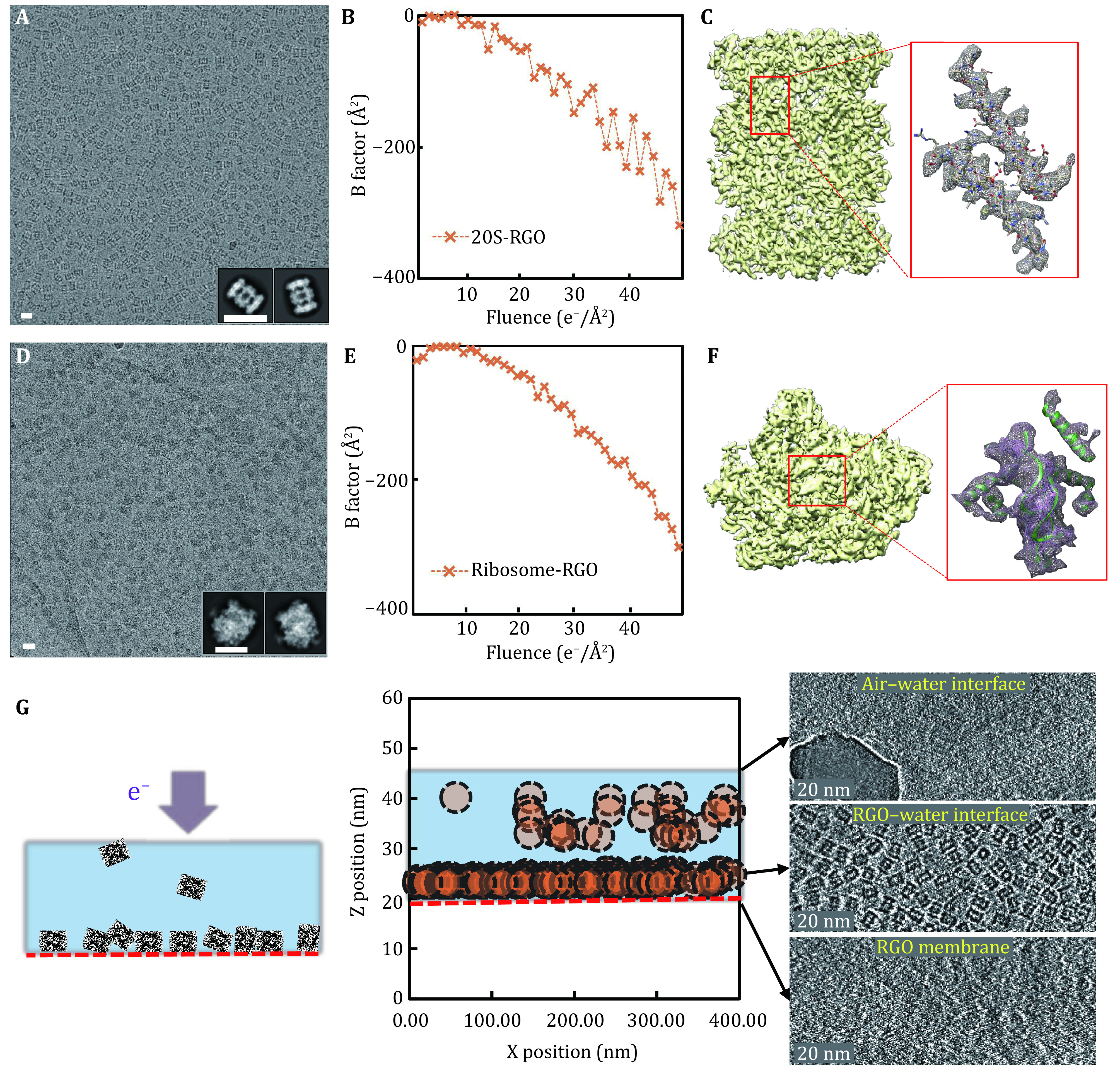
RGO for cryo-EM reconstructions. **A** A representative micrograph of 20S proteasome on RGO membrane. The inset was the two-dimensional classification results. Scale bar represented 20 nm. **B** The B factor of the reconstruction of 20S proteasome on RGO was plotted with the accumulated dose. **C** The reconstructed cryo-EM map of 20S proteasome. The density detail with the corresponding atomic model (PDB: 3J9I (Li *et al*. 2013)) docked was listed aside in the red box. Some side chains could be clearly recognized. **D** A representative micrograph of ribosome on RGO membrane. Scale bar represented 20 nm. **E** The B factor of ribosome plotted with the accumulated dose. **F** The reconstructed cryo-EM map of ribosome. The density detail with the corresponding atomic model (PDB: 5H4P (Ma *et al*. 2017)) docked was listed aside in the red box, consistent with the reported resolution. **G** Cryo-electron tomography of 20S proteasome specimen supported by RGO membrane. Left: the schematic diagram of 20S proteasome particles on RGO membrane, indicated by red dotted line. Middle: the distribution of particles position in ice. The dotted circles indicated individual particles, and the red dotted line indicated RGO membrane. Right: three selected cross sections of the cryo-specimen ET reconstruction. From up to bottom were air–water interface, RGO-water interface and RGO membrane sections, respectively. 20S proteasome particles were mainly distributed on the RGO-water interface

We applied RGO grids to prepare cryo-specimens of 20S proteasome and ribosome, and collected datasets using the same parameters on a Tecnai Arctica microscope (200 kV) with Falcon II camera (Fig. 4). The particles of both biomolecules were distributed as monodispersed and high-contrast particles on RGO supporting membranes (Fig. 4A and 4D). Radiation damage induced by the electron beam is one of the key concerns to be considered in cryo-EM imaging. High-resolution content, *i.e*., the chemical bonds in biomolecules, is hypothesized to be destroyed first by electron dose during EM imaging. B factor is proposed in cryo-EM field to model the dose and radiation damage on high-resolution information of biomolecules. Here, we ploted B factors of proteasome and ribosome supported by RGO grids with accumulated electron dose (Fig. 4B and 4E). The B factors of the first 1–5 e^−^/Å^2^ dose were relatively higher than those of the following 5–10 e^−^/Å^2^, which was mainly resulted from the intial beam-induced motion (Scheres 2016). We calculated the decay rates of B factor after the first 10 e^−^/Å^2^ of proteasome and ribosome, both of which were approximately 8 Å^2^/(e^−^/Å^2^). Notably, the B-factor decay rates were close to the radiation damage measurements of protein 2D crystals (Peet *et al*. 2019).

We also prepared cryo-EM specimens of 20S proteasome and ribosome using GO grids. Interestingly, after processing the three-dimensional reconstruction, the Euler angle distribution of particles on RGO was slightly different from that on GO. There were certain portion of 20S proteasome particles with top view (circle shape) on GO membrane (supplementary Fig. S5), which were totally absent on RGO (Fig. 4A). Since 20S proteasome is highly symmetrical, these side-view projections on RGO were enough to reconstruct its structure. We finally obtained the 20S proteasome reconstruction at 4.4 Å resolution on RGO and 4.7 Å resolution on GO, using the same number of particle images. For ribosome sample of no symmetry, distribution of particle orientations was more balanced on RGO membrane (supplementary Fig. S6A and S6C), thus enabling us to get a reconstruction of the ribosome with more reliable structural features than that determined by using particles on GO membranes (Fig. 4C, right and supplementary Fig. S6). We further plotted the directional FSC (supplementary Fig. S7) (Tan *et al*. 2017) and calculated the cryoEF value (Naydenova and Russo 2017), which was 0.28 on GO grid, while 0.42 on RGO grid. Both the directional FSC plots and cryoEF values indicated that ribosome particles on RGO adopted richer orientations. Such variation of particle-orientation performance was probably due to the distinct interacting favor of the biomolecular local surface with the functional groups on supporting membranes whose composition and distribution are different between GO and RGO surfaces. The overall resolution of ribosome reconstruction was reported as 6.1 Å on RGO, which was 8.0 Å on GO (supplementary Fig. S8 and S9).

Furthermore, we evaluated the protein particle’s distribution in the vitreous ice on RGO-supported specimen using cryo-electron tomography (cryo-ET) technique and found that the majority of particles were absorbed onto RGO surface, thus avoiding damage by the air–water interface (Fig. 4G). The ice thickness of the tomogram reconstruction was estimated to be ~20 nm, indicative of little extra background noise generated by the ambient ice.

### Cryo-EM analysis of protein molecules of small molecular weight on RGO grid

From the above analysis, we reasoned that the RGO grid should benefit more to the cryo-EM analysis of smaller macromolecules. We therefore further tested the application of RGO grids on cryo-EM imaging of small biomolecules, such as a 60-kDa protein glycosyltransferase (supplementary Fig. S10A) and a 50-kDa protein Rv2466c (supplementary Fig. S10B), both demonstrating particle images of monodisperse and good contrast. Notably, using the RGO grids, Bai *et al*. reconstructed the cryo-EM structure of DEAH-box ATPase/helicase Prp2 (~100 kDa) at a better-than-3-Å resolution, revealing the role of Prp2 in RNA translocation and spliceosome remodeling (Bai *et al*. 2020).

**Figure 5:**
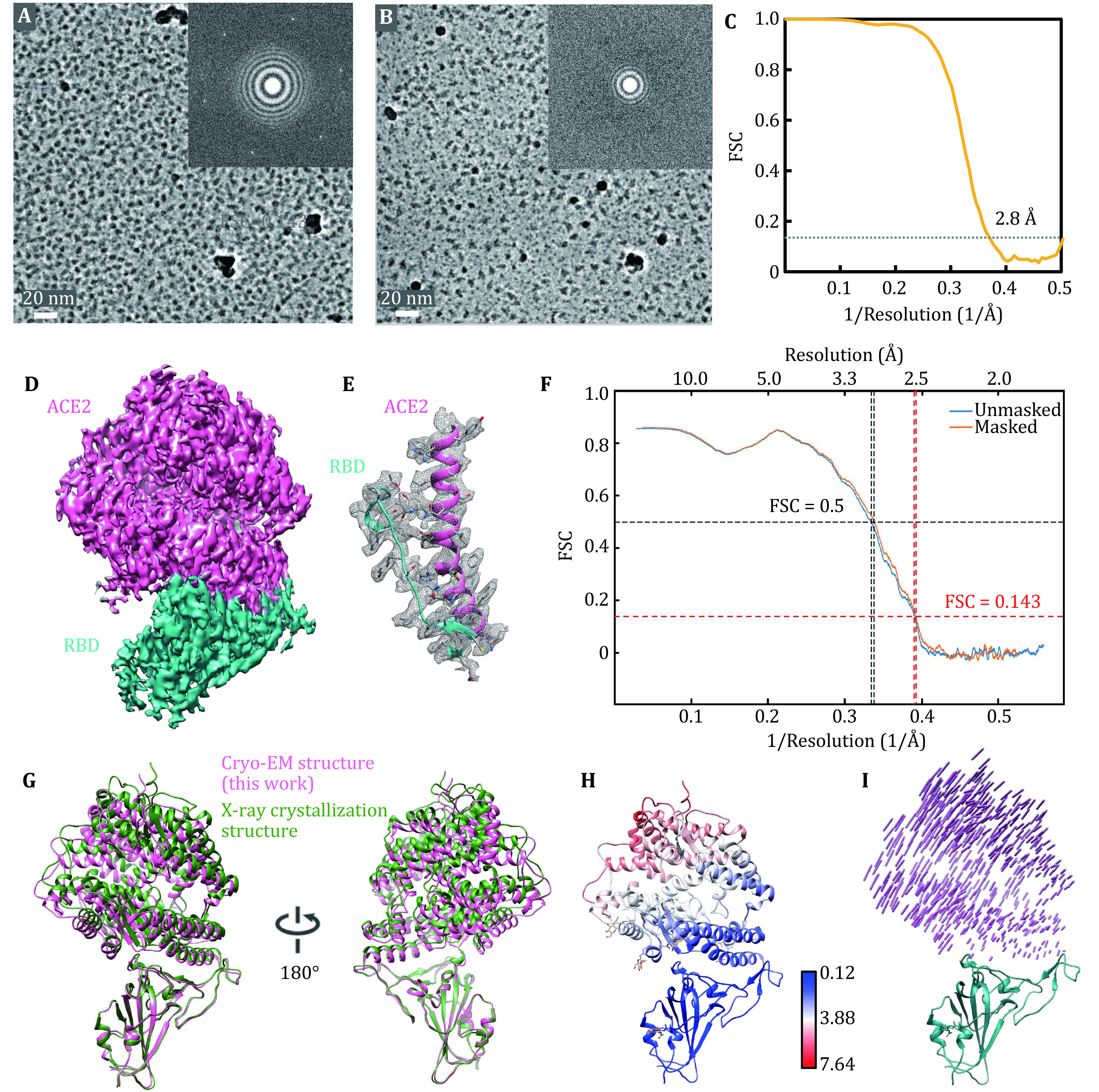
Cryo-EM reconstruction of ACE2-RBD complex supported by RGO membrane. **A** A representative micrograph of RBD-ACE2 complex in the RGO supporting area. The inset was the corresponding FFT image, where six first-order diffraction spots of graphene were sharply displayed. **B** A representative micrograph of RBD-ACE2 complex in the RGO-broken area. **C** The FSC curve of ACE2-RBD complex reconstruction. The dotted gray line indicated FSC = 0.143, which was used for estimating the resolution. **D** The cryo-EM density of ACE2-RBD complex. The pink region was ACE2, while the cyan was RBD. **E** The ACE2-RBD interaction interface with their atomic models docked. **F** The ACE2-RBD model-map FSC curve. **G** Comparison between the cryo-EM structure solved in this work (colored in pink) with the reported X-ray crystal structure (PDB: 6M0J, in green). **H** RMSD (Å) between the cryo-EM structure and the X-ray crystal structure. **I** 3D vector map between the cryo-EM structure and the X-ray crystal structure, using RBD as the alignment reference. The vector lengths indicated the displacement scale across these two structures

COVID-19 virus was identified as a novel pathogenic coronavirus emerging to be an enormous threat to the global public health. Receptor binding domain (RBD) of its spike protein exhibited high binding affinity to the ACE2 receptor on human cells (Zhou *et al*. 2020). We applied RGO grids to prepare the cryo-specimen of RBD-ACE2 complex (95 kDa) and can unambiguously recognize monodispersed particles with good density and contrast on RGO-film-covered area where the graphene diffraction spots were clearly revealed in the Fourier transform (supplementary Fig. S11A). The contrast of protein particles absorbed on RGO film was further improved when utilizing volta phase plate in cryo-EM (Fig. 5A). In contrast, in the area where RGO film was broken (*i.e*., without RGO membrane support), the RBD-ACE2 particles density was significantly decreased and many extra noisy spots of smaller size compared with target particles were observed in the background (Fig. 5B and supplementary Fig. S11B), probably composed of denatured proteins or dissociated components due to the air–water interface interaction (Fig. 4G). From these particle images, we were able to obtain reconstruction of the RBD-ACE2 complex at 2.8 Å resolution and build the atomic model of RBD-ACE2 complex (Fig. 5C–F). The interaction interface of ACE2 and RBD of spike protein was clearly recognized in the cryo-EM density (Fig. 5E). Interestingly, we found that the cryo-EM structure was more compact than the previously reported structure of the same complex solved by X-ray crystallography (Fig. 5G–I) (Lan *et al*. 2020). There are two possibilities that might lead to such difference. One possibility is that a distinct conformation is favored in the crystal packing of RBD-ACE2 complex during crystallization. While in cryo-EM reconstruction, particles are supposed to be in solution, thus adopting a different conformation. The other possibility is that the adsorption of RBD-ACE2 particles to RGO surface might trap the complex at a specific state. Taken together, the RGO grid was suitable for better protein preservation and cryo-EM analysis of some small biomolecules.

## CONCLUSION

Graphene membrane has long been explored as the supporting film in electron microscopy (Wilson *et al*. 2009). The sp^2^ carbon-atom crystalline lattice enables graphene ultrahigh electrical/thermal conductivity. The single-atomic thickness much smaller than the mean free path of electron presents negligible background noise. Yet its practical application is largely limited by the difficulty of reproducible high-quality graphene synthesis and free-of-contamination transfer onto EM grids. Here we described a robust method to fabricate single- or few-layer reduced graphene oxide membrane coated EM grids in large scale. We demonstrated that RGO contained decreased interlayer space and fewer functional groups, compared with GO. Importantly, we found that RGO can recover the electrical conductivity to a certain extent and the capability of reducing charging effects, thus improving the image stability. RGO was able to keep particles on its surface and more friendly than the air–water interface. We finally applied RGO grids for cryo-EM specimen preparation of sub-100-kDa protein samples to reconstruct the small molecules at near atomic resolution. What needs to be pointed out here is that adsorption of particles onto the surface of graphene supporting film often resulted in preferred orientation or other unfavorable consequences that still limited the resolution that could be achieved. Better reconstruction results might be obtained by using RGO with multiple bioactive functionalization.

## MATERIALS AND METHODS

### Preparation of graphene oxide solution

The graphene oxide solution was purchased from Sigma-Aldrich company (Lot #777676) or prepared by improved Hummer’s method (Marcano *et al*. 2010). Briefly, 9 g KMnO_4_ and 1.5 g graphite flakes were added and mixed into 200 mL acid solvent (H_2_SO_4_/H_3_PO_4_ with a volume ratio of 9:1). The mixed solution was then stirred for 12 h at 50 °C. Afterwards, the solution was cooled down in ice bath and was added with 200 mL ice-cold water containing 3 mL H_2_O_2_. After 2-h standing on ice, the mixture was centrifuged at 5000 *g* for 20 min, and the precipitate was carefully collected and washed, and finally resuspended in distilled water to make graphene oxide (GO) solution.

### Fabrication of GO grids

Graphene oxide membrane was transferred onto Quantifoil gold EM grids by a similar procedure described by Palovcak *et al*. (Palovcak *et al*. 2018). Briefly, the GO solution was first mixed into the dispersant (methanol/water with a volume ratio of 5:1) and sonicated for 10 min. The solution was next centrifuged at 4000 *g* for 10 min and the pellets were resuspended in the dispersant solution, followed by a 2-min sonication. Then the GO solution was centrifuged at 500 *g* for 1 min, and the supernatant was carefully collected for coating EM grids. When fabricating GO EM grids, we firstly submerge a steel mesh stand covered by a piece of filter paper into a water-filled container with outlets at its bottom (supplementary Fig. S1B). The Quantifoil gold EM grids were mildly glow-discharged in advance and put onto the steel stand. GO solution was gently pipetted onto the water bath surface to form GO film and the water was slowly drained to lay the GO film onto the grid surface. Finally, the EM grids supported by the filter paper were carefully taken out from the container and air-dried at a 60 °C baker.

### Preparation of RGO grids

The as-fabricated GO grids were placed in a quartz boat and put into a clean tube furnace at room temperature. A flow of 100 sccm H_2_ and 100 sccm Ar was introduced to drain the air out of the furnace. The GO grids were then heated to 300 °C at a rate of 2 °C/min, under the atmosphere of 100 sccm H_2_ and 100 sccm Ar. Subsequently, the grids were reduced at 300 °C for 1 h under the same atmosphere. After reduction, the grids were naturally cooled down to room temperature and ready for use.

### Characterization

The morphologies of the GO and RGO membranes were characterized using AFM (Bruker dimension icon, scansyst mode, scansyst air tip). The composition characterizations of GO and RGO membranes were conducted with XPS (Kratos Analytical AXIS-Ultra with monochromatic Al Kα X-ray). The four-probe resistance measuring meter (CDE ResMap 178) was used to measure the sheet resistances of GO and RGO membranes on mica substrates. The graphene layer number was determined by the diffraction pattern under TEM. The first-order diffraction pattern of single-layer graphene contained six spots, the density of which were similar with that of the second-order diffraction spots. The water contact angle of RGO and GO grids after plasma treatment was measured by the optical contact angle measuring device (Dataphysics company).

### The sheet resistance measurement

To measure the sheet resistance, four-point probes were equally spaced and arranged in a line on the target material. By measuring the current (*I*) through the outside two probes and the voltage (*V*) across the inside two probes, the average sheet resistance (*R*_s_) of the conducting film can be acquired. When the thickness of the conducting film is much less than the spacing of probes, and the width of film is apparently larger than the spacing distance, the average sheet resistance can be given by *R*_s_ = 4.53*V*/*I*.

### Cryo-EM specimen preparation

*Thermoplasma acidophilum* 20S proteasome was recombinantly expressed in *Escherichia coli* cells and purified as described previously (Li *et al*. 2013). Yeast ribosomes were purified from yeast cells following previously published protocols (Ma *et al*. 2017). RBD-ACE2 complex was prepared according to methods utilized in (Lan *et al*. 2020). ~3 μL solution containing purified biomolecules was pipetted onto freshly glow discharged RGO grids, and then transferred into an FEI Vitrobot. For glow discharging, we used low-energy plasma to treat the RGO grids for 15 s in a Harrick PDC-32G plasma cleaner. The humidity of Vitrobot chamber was kept as 100% and the temperature as 8 °C. The grids were then blotted 2 s with –2 force, immediately followed by plunge-freezing into liquid ethane cooled at liquid nitrogen temperature. After that, the grids were quickly transferred into liquid nitrogen for storage.

### Cryo-EM data collection and analysis

The single-particle cryo-EM datasets of 20S proteasome and ribosome were collected on an FEI Tecnai Arctica (200 kV), equipped with a Falcon II detector. The RBD-ACE2 dataset was collected on an FEI Titan Krios (300 kV), equipped with a Gatan K3 summit detector and Volta phase plate, using AutoEMation software (Fan *et al*. 2017; Lei and Frank 2005). All micrographs were dose-dependently fractionated into 32 frames, with an accumulated dose of 50 e^−^/Å^2^. The individual frames were firstly motion-corrected by MotionCor2 algorithm (Zheng *et al*. 2017) and the resulted micrographs were imported into Relion3.0 (Zivanov *et al*. 2018) for further processing. CTF values were calculated by CTFFIND4 package (Rohou and Grigorieff 2015). Biomolecular particles were autopicked and iteratively 2D classified in Relion3.0. Particles grouped in good classes exhibiting fine structural details were selected for further 3D classification and refinement. We used Fourier Shell Correction (FSC) 0.143 cutoff criteria to estimate the resolution in the final postprocessing step in Relion3.0. Finally, we used 10,000 and 8,453 particles for the reconstructions of 20S proteasome and ribosome. The resolution of 20S proteasome and ribosome on RGO grids was 4.5 Å and 6.1 Å, respectively. For RBD-ACE2 complex, we finally used 110,122 particles and got a reconstruction at 2.8 Å resolution. All figures related to cryo-EM structures were created in UCSF Chimera (Pettersen *et al*. 2004). The directional FSC curves were generated in remote 3DFSC processing server (https://3dfsc.salk.edu) (Tan *et al*. 2017).

### Cryo-electron tomography analysis

Cryo-ET micrographs series were obtained on an FEI Titan Krios microscope (300 kV), equipped with a Gatan K2 camera. We used SerialEM (Mastronarde 2005) to collect tilt series, and the specimen was tilted from +51° to –51° with an acquired step of 3°. The total dose was 100 e^−^/Å^2^ with a dose rate of ~3 e^−^/Å^2^/s, and the calibrated pixel size was 1.77 Å. The micrographs series was imported into IMOD (Kremer *et al*. 1996) for reconstruction, where the position of protein particles was manually identified as previously (Liu *et al*. 2019).

### Data and material availability

The cryo-EM map of SARS-CoV2 RBD-ACE2 complex has been deposited in the EMDB under accession number EMD-30816, and the coordinate in the PDB with PDB ID 7DQA.

## Conflict of interest

Nan Liu, Liming Zheng, Jie Xu, Jia Wang, Cuixia Hu, Jun Lan, Xing Zhang, Jincan Zhang, Kui Xu, Hang Cheng, Zi Yang, Xin Gao, Xinquan Wang, Hailin Peng, Yanan Chen and Hong-Wei Wang declare that they have no conflict of interest.
